# Perforating scleral vessel angular metrics as a structural biomarker of myopic choroidal neovascularisation

**DOI:** 10.1038/s41433-026-04781-x

**Published:** 2026-08-01

**Authors:** Alberto Quarta, Alessandro Feo, Francesco Romano, Giulia Corradetti, Federico Corvi, Mai Alhelaly, Rouzbeh Abbasgholizadeh, Shinichiro Chujo, Marko Popovic, Ceren Soylu, Yu-Chien Chung, Hyun Duck Kwak, Jianfeng Huang, Muneeswar G. Nittala, Giovanni Staurenghi, Srinivas R. Sadda

**Affiliations:** 1 Doheny Eye Institute, Pasadena, CA USA; 2 Department of Ophthalmology, David Geffen School of Medicine, University of California, Los Angeles, CA USA; 3 Department of Neurosciences, Imaging and Clinical Sciences, University “G. d’Annunzio” Chieti-Pescara, Chieti, Italy; 4 Eye Clinic, Department of Biomedical and Clinical Science “Luigi Sacco”, Sacco Hospital, University of Milan, Milan, Italy; 5 Ophthalmology Department, Tanta University, Tanta, Egypt; 6 Department of Ophthalmology, Mie University Graduate School of Medicine, Tsu, Japan; 7 Department of Ophthalmology, Fu Jen Catholic University Hospital, Fu Jen Catholic University, New Taipei City, Taiwan; 8 Beijing Hospital, National Center of Gerontology, Institute of Geriatric Medicine, Chinese Academy of Medical Sciences, Beijing, PR China

**Keywords:** Predictive markers, Biomarkers, Eye manifestations

## Abstract

**Background:**

To quantitatively assess the entrance angle variability and location of perforating scleral vessels (PSVs) in highly myopic eyes and to investigate its association with myopic choroidal neovascularisation (mCNV) versus lacquer cracks (LC).

**Methods:**

Entrance angles from the sclera to choroid were measured on each B-scan OCT using ImageJ and associated angle metrics were calculated per PSVs. Additional variables included subfoveal choroidal thickness (SFCT) and vessel-to-fovea distance (VFD). In mCNV eyes the total number of injections and treatment duration were also accounted.

**Results:**

A total of 42 eyes were included, 25 with mCNV and 17 with LC. Eyes with mCNV demonstrated significantly greater PSV angular variability including angular standard deviation, and their normalised metrics compared to LC eyes (all *p* < 0.001). VFD was shorter in the mCNV group (50.2 ± 52.6 µm vs. 91.5 ± 39.6 µm, *p* = 0.006). Normalised angle metrics correlated positively with the total number of anti-VEGF injections (*r* ≈ 0.37, *p* < 0.05), whereas no association was observed with treatment duration. In multivariable regression, no predictors reached statistical significance although normalised angle range (NAR) showed a trend toward a negative association with VFD (*p* = 0.060).

**Conclusions:**

PSV angular variability was markedly greater in mCNV eyes and correlated with anti-VEGF injection burden reflecting higher treatment intensity but not longer disease duration. These findings suggest that PSV geometry influences LC and mCNV, may serve as an imaging biomarker of disease activity in mCNV. Shorter VFD and greater angular dispersion likely reflect structural and hemodynamic alterations predisposing to mCNV.

## Introduction

Pathologic myopia is a predominant cause of irreversible vision impairment globally, particularly in East and Southeast Asia where its prevalence continues to rise [1]. One of its most common vision-threatening complications is myopic choroidal neovascularisation (mCNV), which may result in central vision loss despite available anti-VEGF therapies [2,3].

Recent research has pivoted attention toward the role of perforating scleral vessels (PSVs) in the development and progression of mCNV [4–6]. In a multicentre cohort of highly myopic eyes, Ruiz-Medrano et al. reported PSVs in 93.5% of eyes with mCNV and notably, about 80% of those PSVs were situated directly under or immediately adjacent to neovascular lesions—indicating a potential anatomical predisposition for mCNV development in proximity to PSVs [4]. Yao et al. documented that eyes with PSVs adjacent to mCNV demonstrated poorer outcomes following anti-VEGF treatment characterised by less visual acuity improvement and smaller reductions in macular thickness [5]. Further corroborating these observations, She et al. found that the presence of PSVs adjacent to mCNV increased the risk of lesion recurrence [7].

Building on these findings, more recent work by Ruiz-Medrano et al. (2025) using OCTA showed PSVs and dilated choroidal veins (DCVs) are prevalent in high myopia–regardless of the presence of mCNV–suggesting that their mere presence may not be specific marker for risk, but rather their anatomical configuration and behaviour may confer higher pathogenic relevance likely reflecting choroidoscleral remodelling [6]. Beyond mCNV other works have demonstrated the link between PSV and lacquer cracks (LC) in a similar fashion [8,9].

Despite this growing body of literature, there is a critical gap in the quantitative assessment of PSVs geometry – specifically in describing their trajectory, spatial variability, and relationship to the fovea [10]. We hypothesised that the angle which PSVs take through the sclera to enter the choroid may influence the biomechanical impact of these structures and could potentially affect the development of mCNV and its response to therapy [9,11].

In this study, we evaluated PSVs in eyes with mCNV versus LC, quantifying their angular metrics and spatial relationship to the fovea aiming to understand whether PSV orientation can serve as an imaging biomarker for mCNV susceptibility and activity.

## Methods

### Study design and participants

This retrospectively collected cross-sectional analysis included patients with a diagnosis of mCNV or LC who underwent multimodal imaging at Ospedale Luigi Sacco (Milan, Italy) and the Doheny Eye – UCLA Eye Centers (Pasadena, CA, USA) between January 2021 and January 2025. The study adhered to the principles of the Declaration of Helsinki, complied with the Health Insurance Portability and Accountability Act (HIPAA) and Italian bioethics guidelines, and was granted a waiver of informed consent due to its retrospective design.

### Study population and procedures

All subjects were required to have undergone a comprehensive ophthalmic evaluation including detailed medical history collection, best-corrected visual acuity (BCVA) assessment, and biomicroscopy in addition to retinal imaging. The diagnosis of mCNV was confirmed via multimodal imaging requiring at least one among optical coherence tomography (OCT), OCT angiography (OCTA), fluorescein angiography (FA) or indocyanine green angiography (ICGA) as previously described [2]. LC were defined as linear or stellate lesions without hyperpigmented margins and not co-localising to choroidal excavations, requiring at least one among OCT, infrared reflectance (IR), fluorescein angiography (FA) or fundus autofluorescence (FAF) for diagnosis as previously described [8]. Inclusion criteria for each group were availability of Spectral-domain (SD) OCT at the first visit with B-scan density of 49–97 B-scans/6 mm, 20 × 20° (Spectralis OCT2, Heidelberg Engineering GmbH, Heidelberg, Germany), and absence of other retinal vascular disorders or degenerative maculopathies, absence of chorioretinal atrophy in correspondence with the mCNV or LC. Exclusion criteria for both the mCNV group and the LC group included poor image quality (SSI < 7), prior photodynamic therapy or anti-VEGF injections, myopic stretch lines (defined as pigmented brown linear lesions that appear hypofluorescent on fluorescein angiography and correspond to clumps of retinal pigment epithelium on OCT) [12], history of ocular inflammation; significant media opacities and coexisting chorioretinal diseases (e.g., vein occlusion, diabetic retinopathy, angioid streaks, macular dystrophy). Myopic maculopathy was graded using the ATN classification system proposed by Ruiz-Medrano et al. [13]. For eyes with mCNV the number of intravitreal anti-VEGF injections administered during the follow-up period until the last visit was documented. The total number of injections was defined as the cumulative count of anti-VEGF treatments administered during the observation period reflecting the overall therapeutic burden, as defined in previous studies [14,15]. The treatment duration was calculated as the time interval (in months) between the first and the most recent injection [16–18]. Therapeutic response was defined based on resolution of intraretinal, subretinal fluid and subretinal hyperreflective material (SHRM) regression as previously described [19,20].

### PSV identification and measurement of angular metrics

**Fig. 1:**
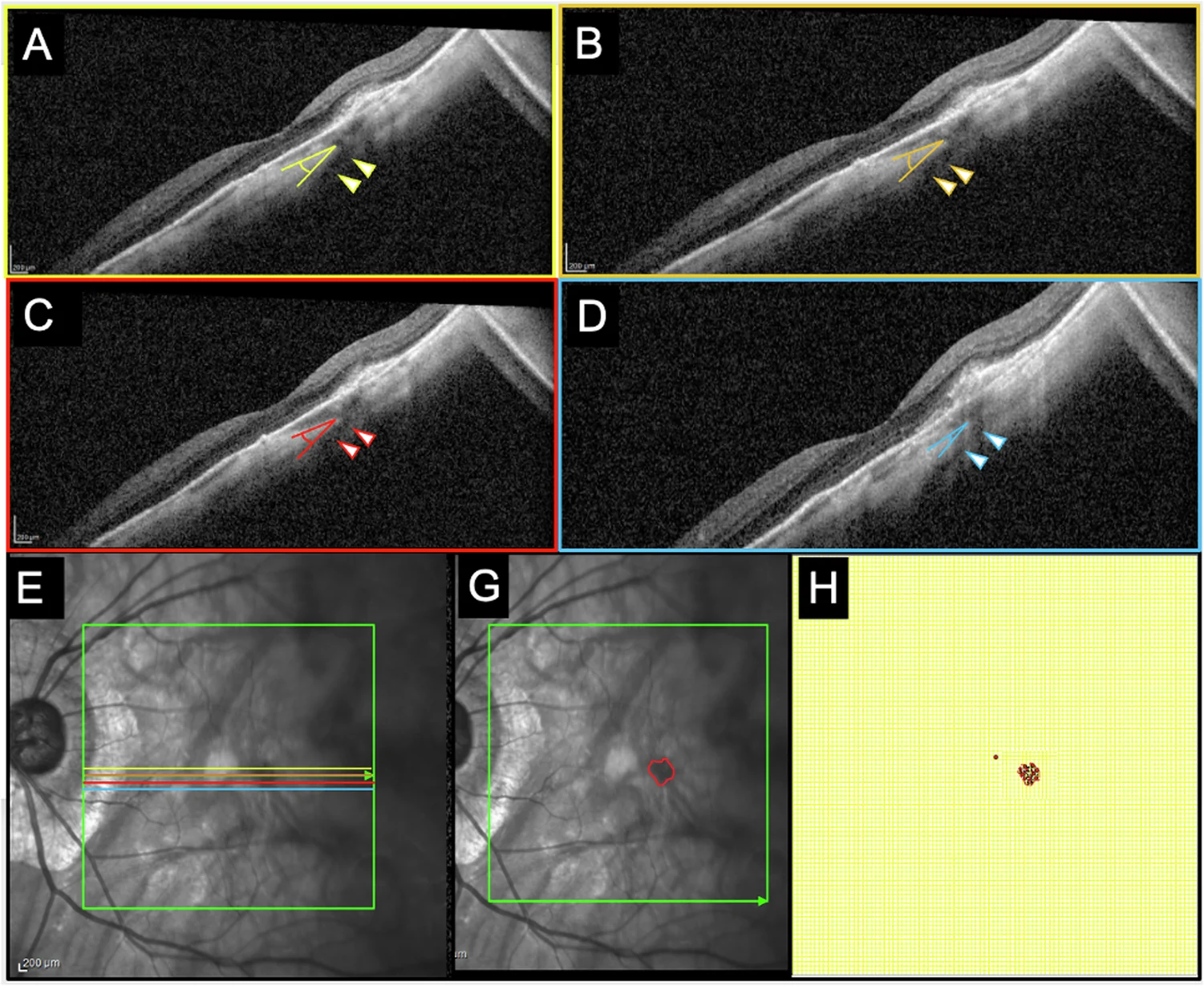
Structural and spatial characterisation of a perforating scleral vessel in a highly myopic eye with mCNV. **A**–**D** Cross-sectional optical coherence tomography (OCT) B-scans illustrating the entrance of a perforating scleral vessel (indicated by coloured arrowheads) traversing from the sclera into the choroid in close proximity to a myopic choroidal neovascularisation (mCNV). The vessel’s trajectory displays a characteristic angular or oblique entry as it penetrates the sclera–choroid interface. **A**, **B** Yellow/orange arrows highlight the scleral vessel lumen emerging through the posterior sclera, demonstrating continuity toward the choroidal vasculature adjacent to the CNV. **C**, **D** Red and cyan arrows further delineate the same vessel in consecutive scan sections, emphasising the focal disruption of the scleral–choroidal boundary and the transition zone where the perforating vessel enters the choroid. **E** Corresponding en face structural OCT image showing the exact localisation of the B-scan planes (yellow, orange, red, and blue lines) within the volumetric pattern. **G** Infrared (IR) reflectance image of the same area revealing the corresponding perforating scleral vessel (outlined in red). The vessel is readily visible owing to marked choroidal thinning, a typical feature in highly myopic eyes that enhances visualisation of deep choroidoscleral structures. **H** Topographic reference grid used to measure the distance between the perforating vessel and the foveal center, enabling quantitative assessment of its spatial relationship to the macular region.

PSVs were identified through a combined en-face IR and cross-sectional B-scan OCT analysis and only PSVs with a minimum total interscan extent of ≈250 µm were included, corresponding to visualisation on at least ≈2 consecutive slices in a 49-line horizontal B-scan volume and on ≈ 4 consecutive slices in a 97-line horizontal B-scan volume [4,8]. PSVs were first identified on volumetric cross-sectional B-scans as hyporeflective tubular structures piercing through the sclera and entering the choroid. A vessel was defined as a true PSV only if the corresponding B-scan confirmed the presence of a continuous hyporeflective lumen penetrating the sclera and extending into the choroid, thereby excluding spurious hyporeflective signals caused by shadowing, noise, or segmentation artifacts [4,8]. This allowed assessment of the spatial relationship between PSV trajectories and pathological findings in the posterior pole. The entrance angle of the PSV was defined as the acute angle (measured in degrees) between the longitudinal axis of the vessel as it enters the sclera and the tangential line to the curvature of the choroidoscleral junction at the point of entry of the PSV into the choroid on the first evaluable B-scan OCT (Fig. 1). Image analysis was performed using ImageJ (National Institutes of Health, Bethesda, MD). The maximum and minimum angle values were measured across the analysed B-scans to account for potential directional variability along the visible course of the PSV. Thus, the angle was recorded from all B-scans in which the PSV was clearly visible and only well-visualised entry points were included for analysis. We computed two metrics to describe the PSV trajectory characteristics across the B-scans: (i) *Angle Standard Deviation (SD)*, calculated as the standard deviation of the entrance angle across all B-scans with visible PSV segments, in order to quantify the degree of directional variability or “waviness” of the vessel path. The formula was as follows:

$${{{\rm{SD}}}}=\sqrt{\frac{1}{n-1}{\sum }_{i=1}^{n}{({\theta }_{i}-\overline{\theta })}^{2}}$$

where θi represents the entrance angle in the *i-th* B-scan, θˉ is the mean entrance angle across *n* slices, and *n* is the number of B-scans in which the PSV was visible. A higher SD indicates a more irregular or tortuous vessel path, possibly reflecting focal changes in scleral tension or biomechanical remodelling. To assess the maximum directional deviation of the vessel and capture the full span from the most oblique to the most vertical slice, the *Angular Range* of entrance angles was calculated as: (i) *Range=θmax−θmin*. PSV angle metrics were normalised by vessel length (deg/mm) to further mitigate effects of scan density. The following normalised angular parameters are expressed in degrees per millimetre (°/mm) as follows: *(i) Normalised Angle Standard Deviation (NASD)*, representing the PSV’s SD entrance angles normalised to the vessel’s visible length (PSV extension through B-scans), yielding the angular variability per millimetre of PSV path*; (ii) Normalised Angular Range (NAR*), representing the difference between the maximum and minimum entrance angles divided by the PSV extent through B-scans reflecting the maximum directional variation per unit length.

#### PSV to fovea distance

The anatomical association of PSVs with LC and mCNV was systematically evaluated, recording whether each vessel was located adjacent to or directly beneath LC or mCNV lesions following previously described imaging criteria [4,8]. The PSV coordinate used for VFD measurement was defined primarily by the choroidoscleral entry point confirmed on structural OCT B-scans. The corresponding en face IR image was then used for spatial registration and binary mask generation. Thus, the visible IR vessel centre was not used independently unless it corresponded to the OCT-confirmed PSV entry point. To adjust for PSV distance from the foveal centre in both groups we performed a pixel to millimetres Euclidean distance analysis. The IR en-face slab in which the PSV were clearly visible from both groups were converted into binary masks. The masks were overlaid with a calibrated square grid, dividing the lesion area into contiguous 0.1 mm² units. The foveal centre was manually identified on structural B-scans as the point of maximum foveal depression or, in cases where the fovea was involved by the myopic lesion, based on other available landmarks. The centre of the PSV at the point of entry into the choroid was marked, and the Euclidean distance between each PSV centre and the foveal centre was then computed to assess spatial distribution relative to the fovea.

Considering Xi,Yi = coordinates of each grid point and X0,Y0 = coordinates of the foveal centre, the Euclidean distance was calculated as:

$${d}_{i}=\sqrt{{({X}_{i}-{X}_{0})}^{2}+{({Y}_{i}-{Y}_{0})}^{2}}$$

The mean of all such distances was then computed to obtain the VFD (d).

$$\bar{d}=\frac{1}{N}{\sum }_{i=1}^{N}{d}_{i}$$

To facilitate coordinate-based spatial analysis, VFD values were decomposed into X, Y coordinates. The described method allowed for topography-independent quantification of lesion eccentricity relative to the fovea. All the quantitative metrics were performed by two graders (AQ, AF) with discrepancies resolved by a third senior specialist (SS); the mean between the two measurements was used for statistical purposes.

#### Statistical analysis

Descriptive statistics are presented as mean ± standard deviation (SD). The normality of continuous variables was assessed using the Shapiro–Wilk test. Between-group comparisons were conducted using the *t* test or Mann–Whitney *U* test for continuous variables, and the chi-square or Fisher’s exact test for categorical variables as appropriate. Intergroup differences were assessed using two-sample *t* tests, with Benjamini–Hochberg correction applied for multiple comparisons. Correlation between angle metrics and treatment variables was assessed using Spearman correlation. A multivariable linear mixed-effects model assessed the effect of NAR on VFD, adjusting for age, SFCT and diagnostic group (mCNV vs. LC). Patient ID was included as a random effect to account for intra-individual variability where applicable. Variance inflation factor (VIF) was used to assess multicollinearity. A two-sided *p* value < 0.05 was considered statistically significant. Analyses were conducted in R version 4.1.0 (R Foundation for Statistical Computing, Vienna, Austria).

## Results

### Patient characteristics

A total of 42 eyes were included in the study including 25 eyes with mCNV and 17 eyes with LC. The two groups were comparable in terms of age (mean ± SD: 71.41 ± 12.90 years in mCNV vs 74.24 ± 12.02 years in LC; *p* = 0.626). In the mCNV group (*n* = 25), 8 patients were male (32%) and 17 female (68%). In the LC group (*n* = 17), 6 patients were male (35.3%) and 11 female (64.7%), and differences in sex distribution between the two groups was not significant (*p* = 1.0) (Table 1). For baseline ATN grading, mCNV group showed A1T0N2a (16/25, 64.0%), A2T0N2a (8/25, 32.0%), and A3T0N2a (1/25, 4.0%). In LC group, all eyes were assigned an N1 grade (per definition), distributed across A1T0N1 (10/17, 58.8%), A2T0N1 (4/17, 23.5%), and A3T0N1 (3/17, 17.6%). SFCT was significantly greater in the mCNV group than in the LC group (230.4 ± 61.2 μm and 163.7 ± 59.1 μm, *p* < 0.001). Across treated mCNV eyes, the total number of injections ranged from 2 to 23 (11.3 ± 6.7). The treatment duration spanned from 3 to 75 months (mean 35.7 ± 19.6 months).

**Table 1:** Descriptive statistics for continuous variables in mCNV and LC groups.

Variable	Group	Mean ± SD	*p*-value
Age	LC	74.24 ± 12.02	0.626^a^
	mCNV	71.41 ± 12.90	
VA (logMAR)	LC	0.592 ± 0.550	0.016^a^
	mCNV	0.272 ± 0.320	
Min Angle (°)	LC	14.35 ± 9.55	0.990^a^
	mCNV	14.29 ± 9.78	
Max Angle (°)	LC	59.42 ± 26.19	0.305^a^
	mCNV	66.53 ± 27.18	
Mean PSV Angle (°)	LC	38.24 ± 15.83	0.505^a^
	mCNV	40.41 ± 16.43	
Angle range (°)	LC	2.88 ± 2.44	<0.001^a^
	mCNV	12.82 ± 12.21	
NASD (°/mm)	LC	5.75 ± 4.57	<0.001^b^
	mCNV	52.79 ± 59.82	
NAR (°/mm)	LC	8.15 ± 6.46	<0.001^b^
	mCNV	74.28 ± 84.83	
Angle SD (°)	LC	11.78 ± 7.63	<0.001^b^
	mCNV	20.64 ± 7.13	
SFCT (µm)	LC	163.7 ± 59.1	<0.001^a^
	mCNV	230.4 ± 61.2	
VFD (um)	LC	91.52 ± 39.57	0.006^b^
	mCNV	50.24 ± 52.64	

Variables were compared between groups using the Mann–Whitney U test^a^ for non-normally distributed continuous variables and the Student’s *t* test^b^ for normally distributed continuous variables; all p values were adjusted for multiple comparisons.

*VA* Visual Acuity, *SFCT* Subfoveal Choroidal Thickness, *VFD* Vessel to Fovea Distance, *NAR* Normalised Angle Range, *NASD* Normalised Angle Standard Deviation.

### PSV angular metrics and characteristics

**Fig. 2:**
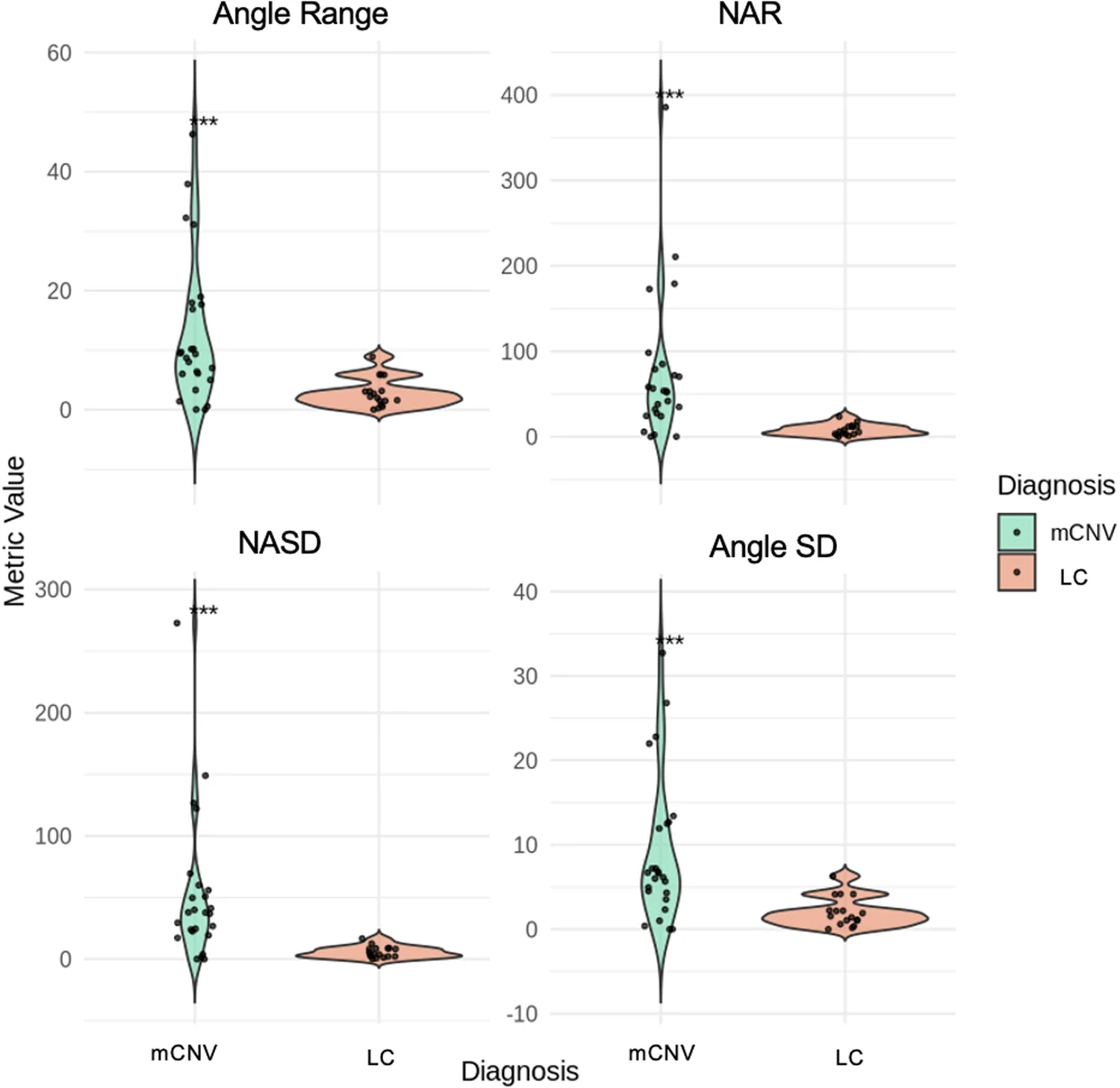
Violin plots showing angular metrics across groups. AngleRange (raw Max–Min) is substantially larger in myopic choroidal neovascularisation (mCNV) (mean ≈ 12.8°) than in lacquer cracks (LC) (mean ≈ 2.9°), with both tests *p* ≤ 0.001. NAR (Normalised Angle Range, deg/mm) remains much higher in mCNV (≈74 deg/mm) vs. LC (≈8 deg/mm), again highly significant. Angle SD (raw dispersion) and NASD (Normalised Angle SD) likewise show strong group differences (all *p* ≤ 0.001).

Although minimum and maximum vessel angles did not differ significantly between groups, derived angle metrics showed marked differences. Specifically, angle range, NAR, angle SD and NASD were all significantly greater in mCNV eyes compared to LC eyes (*p* < 0.001 for all) (Fig. 2). VFD was significantly shorter in mCNV eyes (50.24 ± 52.64 µm vs. 91.52 ± 39.57 µm, *p* = 0.006) (Table 1).

### Correlation analyses

**Fig. 3:**
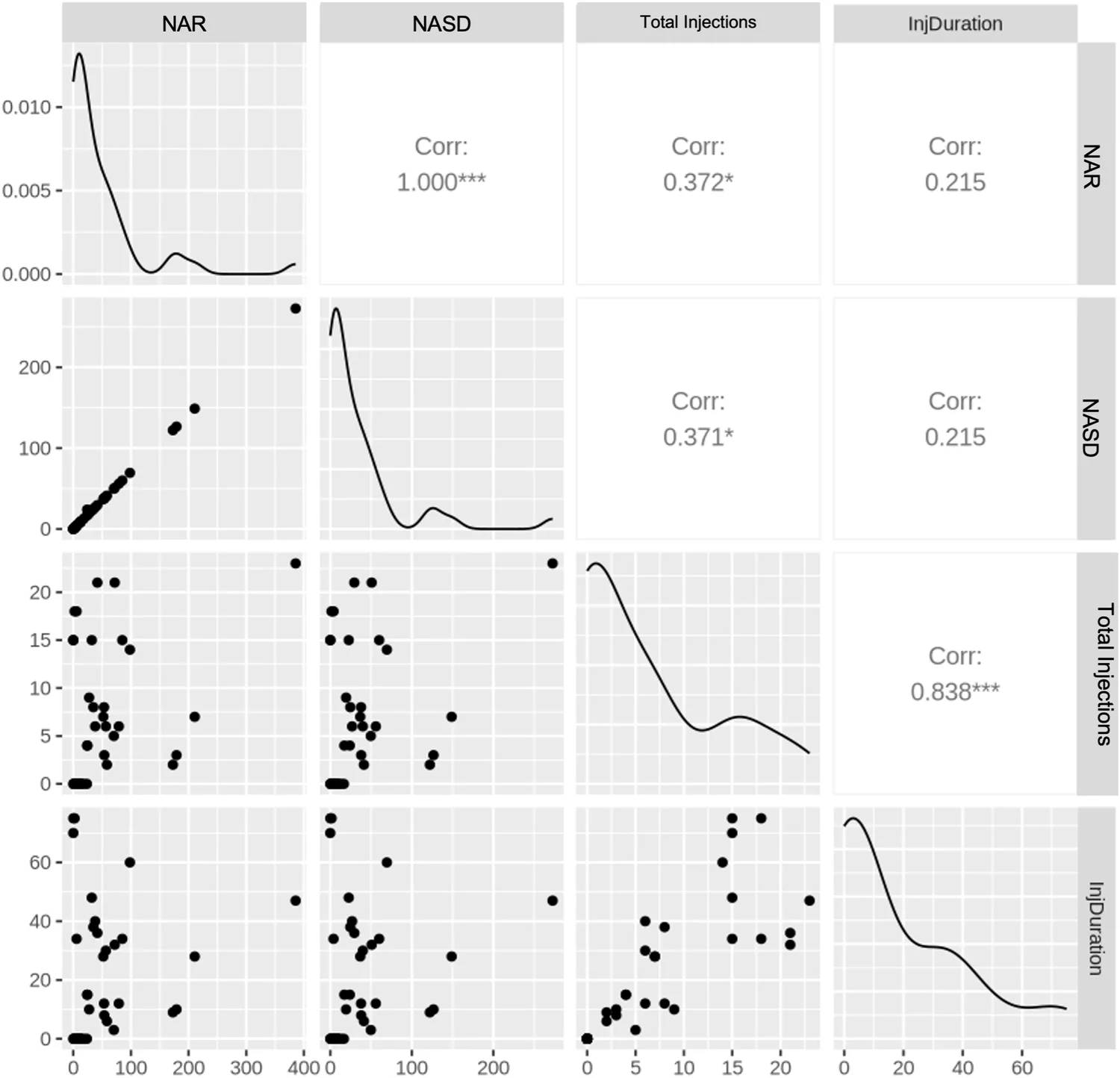
Diagonal (Density Plots) NAR (NormAngleRange) and NASD (Normalised Angle SD). Both plots show right-skewed distributions: most eyes cluster at lower normalised values, with a tail of higher values. Total Injections has a slight right skew (some eyes required many injections). Off-Diagonal (Scatterplots): Upper-left and lower-right panels for Normalised vs. Total Injections show clear upward trends: points slope upward, indicating that eyes with higher normalised angles tend to have received more injections. The corresponding correlation coefficients (*r* ≈ 0.37, *p* < 0.02) confirm these associations as statistically significant. Panels for Normalised vs. Injection Duration are much more diffuse: no clear slope, reflecting weak, non-significant correlations (*p* ≈ 0.17).

Pairwise correlation plots revealed that normalised angle metrics (NAR and NASD) were positively associated with the total number of injections received (*r* = 0.372 and *r* = 0.371, respectively; *p* < 0.05). As expected, treatment duration correlated strongly with total number of injections (*r* = 0.838, *p* < 0.001) but was not associated with angle parameters (Fig. 3).

### Multivariable regression

A mixed-effects linear regression model was applied to predict VFD. Age, diagnostic group (LC vs mCNV), NAR, and SFCT were included as predictors. None reached statistical significance, although NAR showed a trend toward a negative association with VFD (B = –0.209, 95% CI [–0.42, 0.00], *p* = 0.060) (Table 2).

**Table 2:** Multivariable linear regression mixed effect model predicting VFD.

Predictor	Estimate	CI	*P* value
(Intercept)	83.858	[−7.53, 175.24]	0.084
Age	−0.253	[−1.40, 0.90]	0.670
Group(mCNV)	26.928	−6.98, 60.84]	0.127
NAR	−0.209	[−0.42, 0.00]	0.060
SFCT	0.037	[−0.52, 0.60]	0.896

*NAR* Normalised Angle Range, *SFCT* Subfoveal Choroidal Thickness, *VFD* Vessel to Fovea Distance.

## Discussion

In this study, we quantitatively characterised the entrance trajectory of PSV in eyes with mCNV and LC using angular metrics. Although minimum and maximum angles individually did not differ significantly, secondary metrics–angle range, angle SD and their normalised forms (NAR, NASD)–were consistently and significantly higher in mCNV compared to LC suggesting a more convoluted or tortuous scleral path in mCNV, which we speculate could be related to localised biomechanical weakening of the sclera enabling a more tortuous course. Notably, VFD was also significantly shorter in mCNV suggesting that PSVs in mCNV eyes tend to emerge closer to the foveal centre. We speculate that a closer proximity to the fovea for PSV entry may create biomechanical or hemodynamic stresses, which may increase the likelihood of mCNV [4,6]. Lastly, the positive correlation between normalised angular metrics and anti-VEGF treatment burden suggests that an irregularity or waviness in the PSV trajectory may impact the activity of the CNV lesion. In particular, we speculate that this irregular trajectory may also create a biomechanical or hemodynamic stress on the choroid and Bruch’s membrane, which may facilitate the development of exudative mCNV [21–24]. However, biomechanical stress, scleral stiffness, and choroidal perfusion were not directly measured in this study; therefore, these mechanisms require validation in prospective studies incorporating biomechanical modelling and depth-resolved vascular imaging.

One of the main findings of this study is that the angular variability of PSVs as captured by quantitative angle metrics was markedly greater in eyes with mCNV compared to those with LC, suggesting that PSV orientation in mCNV is less constrained and more irregular, reflecting alterations in sclerochoroidal architecture [9–11,25]. Importantly, PSVs in mCNV eyes were also situated closer to the fovea supporting the concept that neovascularisation preferentially develops where vessel entry points intersect more central retinal regions [6].

The clinical relevance of these anatomical differences is underscored by our correlation analyses. Both NAR and NASD were positively correlated with the total number of anti-VEGF injections suggesting that greater angular variability of PSVs is also linked to higher treatment burden. This highlights PSV geometry not merely as an anatomic descriptor but as a potential biomarker of disease reactivation as suggested by Ruiz-Medrano et al., who showed a potential role of PSV and dilated choroidal veins in favouring neovascular recurrence [26]. Notably, neither NAR nor NASD correlated with treatment duration implying that PSV variability influences treatment intensity rather than the temporal span of disease activity.

Previous multimodal imaging studies supported the spatial coincidence of PSVs and mCNV lesions. Giuffrè et al. described PSVs as “anatomical anchors” for CNV initiation and reactivation [27]. Ruiz-Medrano et al. further demonstrated that a closer proximity of the PSV to the mCNV location is common in high myopia and relates to mCNV activity [4]. Yao et al. reported that PSVs adjacent to mCNV lesions were associated with poorer anti-VEGF outcomes, while She et al. showed that eyes with neovascular lesion encircling a PSV were more likely to recur [5,7]. The present study did not directly quantify perivascular neovascular signal on OCTA and therefore cannot determine whether angular variability is more closely related to simple PSV adjacency, neovascular encirclement of the PSV, or both. We interpret PSV angular variability as a complementary geometric descriptor that may interact with these previously described spatial biomarkers, and future depth-resolved OCTA studies should evaluate these features together.

The REPAIR trial and the RADIANCE trial demonstrated that intravitreal ranibizumab significantly improved visual outcomes compared with sham or photodynamic therapy (PDT), with many patients requiring relatively few injections (average 3–4 in the first year) [20,28]. The MYRROR trial further confirmed the efficacy of aflibercept in Asian populations also showing marked functional gains with limited treatment burden [29]. Collectively, these trials highlight that mCNV tends to respond rapidly and sustainably to anti-VEGF, but a subset of patients still requires repeated injections due to recurrence or persistence. Our study provides insight into the potential characteristics of this more treatment-resistant patient subgroup, proposing PSV angular variability as a structural marker that may help stratify those patients more likely to fall into the “high-burden” subgroup. While pivotal trials primarily focused on visual acuity and lesion activity on OCT they did not account for underlying biomechanical or vascular geometry and PSV morphology could complement trial-validated treatment protocols potentially serving as an imaging biomarker to predict treatment outcomes. Moreover, whereas the RADIANCE and MYRROR trials emphasised pro re nata (PRN) regimens based on disease activity, PSV metrics may identify eyes at risk of recurrence *before* overt activity manifests supporting a more individualised surveillance strategy. If validated longitudinally, PSV geometry could help bridge the gap between the relatively low injection frequency reported in trials and the higher treatment burden often observed in real-world settings, where recurrent mCNV is not uncommon [30].

Mechanistically, PSV angular variability may represent a geometric fingerprint of posterior pole remodelling. Quantitative OCTA studies have shown progressive microvascular rarefaction and enlarging choriocapillaris flow deficits with increasing axial length [31,32]. Three-dimensional OCT has visualised PSV entry points in vivo and emphasised their relationship to outer retinal perfusion [10]. Biomechanical studies further suggest that scleral stiffness and curvature are regionally heterogeneous in high myopia [11] creating localised stress environments that may promote mCNV development. In this framework, PSV irregularity may not be incidental but a manifestation of biomechanical and vascular remodelling that drives lesion location and recurrence in high myopia. The mixed-effects regression model for VFD prediction did not identify significant predictors although NAR showed a trend toward a negative association suggesting that greater angular variability or tortuosity may be linked to PSVs situated closer to the fovea. While preliminary, this observation strengthens the hypothesis that PSV geometry may influence both anatomical distribution and disease behaviour. Although NAR showed a non-significant trend toward a negative association with VFD, this finding should be considered exploratory and hypothesis-generating rather than confirmatory.

The comparison with LC suggests that PSV-related changes may not represent a single uniform pathway. LC and mCNV may be viewed as related but distinct manifestations within the broader biomechanical spectrum of pathologic myopia. The greater PSV angular variability observed in mCNV eyes suggests that different PSV configurations may predispose to different phenotypes of myopic maculopathy rather than merely reflecting the presence of PSVs alone.

This study has several limitations. First, the retrospective design and modest sample size limit generalisability, and detailed myopia phenotyping including AXL, posterior staphyloma configuration, and atrophic maculopathy was not available, all of which may influence scleral biomechanics. We also did not directly measure choroidal perfusion or scleral biomechanics in this cohort, which limits our ability to draw mechanistic inferences. Second, subfoveal choroidal thickness differed between groups and could affect PSV geometry. Third, angular measurements relied on B-scans with clearly visible entry points, and the number of analysable scans and vessel length varied across eyes. Additionally, group differences were driven by derived angular dispersion metrics rather than absolute angle values, and potential outlier effects cannot be excluded. Finally, PSV geometry was assessed cross-sectionally; longitudinal stability and temporal changes in relation to disease activity remain unknown. Future multimodal and longitudinal studies will be required to determine whether PSV angular variability predicts incident mCNV or post-treatment recurrence and whether PSV-based metrics can be incorporated into risk stratification frameworks. However, the strong within-group differences between LC and mCNV eyes suggest that PSV geometry contributes information beyond general myopic elongation. We acknowledge that detailed myopia phenotyping, including axial length and posterior staphyloma configuration, was not available for all eyes. This is relevant because staphyloma morphology may alter local scleral curvature and choroidoscleral contour, potentially influencing PSV entrance-angle measurements and derived angular variability metrics. Therefore, the present findings should be interpreted as exploratory, and future studies incorporating axial length, ATN staging, and three-dimensional staphyloma assessment are warranted. Future studies integrating axial length, posterior staphyloma morphology, and PSV geometry will clarify these relationships.

In summary, eyes with mCNV demonstrate PSVs that are both closer to the fovea and demonstrate more angular variability in their course than those with LC. PSV geometry and particularly angular variability was associated with higher anti-VEGF injection burden suggesting its potential as a biomarker of disease severity and treatment demand. These results align with trial data by offering an explanation for interindividual differences in treatment frequency, and support PSV-based metrics as potential imaging biomarkers to refine prognosis, tailor surveillance, and guide therapeutic strategies in mCNV.

### What was known before:

- Perforating scleral vessels (PSVs) are frequently observed in highly myopic eyes and are commonly located adjacent to or beneath myopic choroidal neovascularisation (mCNV) lesions.
- The presence and proximity of PSVs to mCNV have been associated with poorer visual outcomes, higher recurrence rates, and increased anti-VEGF treatment burden.
- However, the mere presence of PSVs lacks specificity, as PSVs are also prevalent in other myopic lesions such as lacquer cracks, and prior studies have largely relied on qualitative or spatial assessments rather than quantitative structural characterisation.

### What this study adds:

- This study introduces quantitative angular metrics of PSVs, derived from OCT B-scans, as a novel structural imaging biomarker in pathologic myopia.
- Eyes with mCNV demonstrate significantly greater PSV angular variability (angle range, SD, and normalised metrics) compared with lacquer crack eyes, suggesting distinct sclerochoroidal biomechanical remodelling. Increased PSV angular variability is associated with higher anti-VEGF injection burden, supporting a link between PSV geometry and disease severity and activity.
- These findings suggest that PSV angular metrics may improve risk stratification, prognostication, and patient selection in future mCNV clinical trials.

## Data Availability

The data that support the findings of this study are not publicly available due to privacy reasons, but are available from the corresponding author upon reasonable request.
